# The association between general practitioner participation in joint teleconsultations and rates of referral: a discrete choice experiment

**DOI:** 10.1186/s12875-015-0261-6

**Published:** 2015-04-21

**Authors:** Tiago Cravo Oliveira, James Barlow, Steffen Bayer

**Affiliations:** Research Associate, Imperial College Business School, Imperial College London, South Kensington Campus, London, SW7 2AZ UK; Chair in Technology and Innovation Management, Imperial College Business School, Imperial College London, South Kensington Campus, London, SW7 2AZ UK; Assistant Professor, Program in Health Services & Systems Research, Duke-NUS Graduate Medical School, 8 College Road, Singapore, 169857 Singapore

**Keywords:** Teleconsultation, Telemedicine, Continuing education, Referral rates, Primary care, Discrete choice experiment

## Abstract

**Background:**

Joint consultations – such as teleconsultations – provide opportunities for continuing education of general practitioners (GPs). It has been reported this form of interactive case-based learning may lead to fewer GP referrals, yet these studies have relied on expert opinion and simple frequencies, without accounting for other factors known to influence referrals. We use a survey-based discrete choice experiment of GPs’ referral preferences to estimate how referral rates are associated with participation in joint teleconsultations, explicitly controlling for a number of potentially confounding variables.

**Methods:**

We distributed questionnaires at two meetings of the Portuguese Association of General Practice. GPs were presented with descriptions of patients with dermatological lesions and asked whether they would refer based on the waiting time, the distance to appointment, and pressure from patients for a referral. We analysed GPs’ responses to multiple combinations of these factors, coupled with information on GP and practice characteristics, using a binary logit model. We estimated the probabilities of referral of different lesions using marginal effects.

**Results:**

Questionnaires were returned by 44 GPs, giving a total of 721 referral choices. The average referral rate for the 11 GPs (25%) who had participated in teleconsultations was 68.1% (range 53-88%), compared to 74.4% (range 47-100%) for the remaining physicians. Participation in teleconsultations was associated with reductions in the probabilities of referral of 17.6% for patients presenting with keratosis (p = 0.02), 42.3% for psoriasis (p < 0.001), 8.4% for melanoma (p = 0.14), and 5.4% for naevus (p = 0.19).

**Conclusions:**

The results indicate that GP participation in teleconsultations is associated with overall reductions in referral rates and in variation across GPs, and that these effects are robust to the inclusion of other factors known to influence referrals. The reduction in range, coupled with different effects for different clinical presentations, may suggest an educational effect. However, more research is needed to establish whether there are causal relationships between participation in teleconsultations, continuing education, and referral rates.

## Background

There has been much debate over the impact of continuing medical education on physician performance and behaviour [1]. While passive didactic strategies – similar to those used in undergraduate medical education – have a negligible impact on performance, there is some evidence that interactive case-based learning may be effective in changing practice, and potentially improving healthcare outcomes [2,3]. Research on adult learning has suggested that education strategies should be organised around contextual real-world situations and reflect practical concerns, as opposed to theoretical abstract subject matter [4,5]. It is thus unsurprising that joint consultations involving patients, general practitioners (GPs) and specialists – such as real-time teleconsultations – have been associated with educational benefits [6], with GPs learning from being present, observing and listening to specialists [7]. The educational benefit is perceived by GPs themselves [8], and may lead them to reflect and change their behaviour [9].

Even though there is scepticism over the role of continuing medical education in improving physician performance, it has been suggested that GP participation in joint consultations may result in better patient management, fewer hospital follow-up appointments, reductions in the number of diagnostic tests and investigations, improvements in health status, reduction in the likelihood of errors associated with sole management by a GP, and fewer referrals to hospitals [10,11]. The impact on referrals has received much attention. It has been reported that a percentage of GP referrals are inappropriate, unnecessary and avoidable [12,13]. Conversely, some suggest that underreferal and late referral might be as important as overreferral [14]. Joint consultations provide opportunities to scrutinise referral decisions, either through direct specialist feedback or indirectly through role modelling and patient-based learning [15]. As GPs become better at identifying patients who need to be referred, problems with overreferral and underreferral should be less important [16,17].

Previous studies have reported a net reduction in referrals as a consequence of GP participation in joint consultations, suggesting that overreferral is the bigger issue. According to estimates from GPs participating in teleconsultations, reductions could be as high as 25% [18,19]. In another study, concerning face-to-face joint consultations, referrals dropped by nearly 50% [6]. While these studies suggest participation in joint consultations may be associated with reductions in rates of referral, they rely on simple frequencies and qualitative estimates which could simply reflect differences in other confounding factors. Decisions to refer are determined by a broad array of factors, from patient characteristics to GP, practice and secondary care factors [20]. Comparing referral rates for different groups of GPs without controlling for other factors may lead to erroneous associations between participation in joint consultations and reductions in rates of referral. It is thus essential to use multivariate models.

Multivariate models are difficult to test empirically due to limited data availability. Observational revealed preference data are rarely available and difficult to collect. When available, they are not usually collected for purposes of assessing the reasons for – or the appropriateness of – referrals. In this case, stated preference methods – such as discrete choice experiments (DCEs) – can provide a number of benefits. First, all participants are presented with the same group of clinical complaints so that differences in their decisions are due to differences in their judgements, not in the information provided about the patients. Second, collecting data through a survey can be significantly cheaper and more feasible than changing the way revealed choice data is routinely collected in practices and hospitals. Third, stated choice methods can be used to disentangle the effects of different attributes and covariates by design, something that is not easy with revealed preference data as some explanatory variables will be highly correlated (e.g., income and education). Fourth, stated choice methods provide information on non-referrals, which is rarely available in observational data. Finally, and perhaps most significantly, these methods can be used to elicit patients and GPs’ preferences separately.

We use the results of a DCE of GPs’ referral decisions in Portugal to explore the association between participation in joint teleconsultations and referral rates, controlling for factors known to influence referral rates such as clinical presentations and waiting times. While DCEs have been used extensively to elicit patient preferences for referrals, this is, to our knowledge, the first time the methodology is used to study the determinants of GP referrals.

## Methods

Joint teleconsultations – real-time outpatient appointments using video-conferencing equipment to connect patients visiting their GPs to remotely located consultants – were first implemented in Portugal in 1998 [21]. By 2011, more than 32,000 teleconsultations had been provided, the majority of which in the Alentejo region. Dermatology accounted for most teleconsultations performed, followed by cardiology and neurology. Other specialties included physical and rehabilitation medicine, respiratory medicine, urology and psychiatry. In Portugal, patients seeking an appointment with a consultant must first be referred by a GP. If the GP practice is part of the telemedicine network then the patient must first be referred to a teleconsultation before a subsequent face-to-face appointment can be arranged, if necessary. Different practices adopt different organisational approaches. Some appoint a GP coordinator who is present in every teleconsultation for a specific specialty; in others, the patient’s own GP is present. There are no financial incentives for GPs to use teleconsultations and while practices are reimbursed for teleconsultations, these add to GP workload so that there is no clear financial incentive to provide these appointments.

### Experimental design and data collection

A DCE is a survey method based on the assumption that a good or a service (e.g., referral to a specialist) can be described by a set of characteristics or attributes (e.g., waiting time), and that the extent to which individuals (e.g., GPs) value that good or service is determined by the nature and levels of the characteristics [22]. Put simply, GPs’ decisions to refer are affected by a number of factors or attributes, and by observing their decisions it might be possible to determine how the factors and attributes affect their choices. The method has been used extensively to elicit patient and physician preferences for healthcare services [23]. Conducting a DCE in the context of GP referrals involves a number of steps [24]: identifying the relevant attributes affecting the decision to refer and their levels; selecting a sample of possible level combinations (i.e., referral choices) to include in a questionnaire; distributing the questionnaire to a sample of GPs; and analysing GPs’ responses using appropriate regression techniques.

We reviewed determinants of GP referrals and identified four categories of potentially influential factors [20,25,26]: patient characteristics (e.g., clinical need and anxiety for a referral), GP characteristics (e.g., years of experience and training) practice characteristics (e.g., size and location), and secondary care factors (e.g., waiting time and perceived quality). Previous evidence suggests that perceived clinical need for a referral is the most important factor in GPs’ decisions to refer. To capture this in our questionnaire, we restricted our analysis to four dermatological presentations, selected from a database of actual GP referrals in a major Portuguese hospital. Among the cases that were most commonly referred, we identified conditions which should be managed in primary care (chronic plaque psoriasis and seborrhoeic keratosis) and conditions which require urgent attention and referral (cutaneous malignant melanoma and melanocytic naevus), so as to cover the spectrum of clinical need and urgency. Textual descriptions were produced for each case with the help of a senior dermatologist and supplemented with photographs of the lesions. Dermatology was chosen for a number of reasons: variation in referral rates to dermatologists has been shown to exist [27], potentially signalling overreferral and/or underreferral; dermatological lesions tend to develop independently of other illnesses [27], so that the potential confounding effect of co-morbidities can be disregarded; and cases can be described in paper form using text and images with relatively little loss of information (as opposed to neurological cases, for example).

Based on the review of determinants of referrals, we further posited that decisions were affected by three other attributes for which levels were chosen so as to reflect the Portuguese context: average waiting time for a dermatology appointment (levels included 30, 60, 150 and 365 days); distance from GP practice to dermatologist (levels were 0, 30, 60 and 100 kilometres); and pressure from patients or families to be referred (levels were yes or no). Patient age and gender were excluded given previously mixed evidence on their impact.

**Figure 1:**
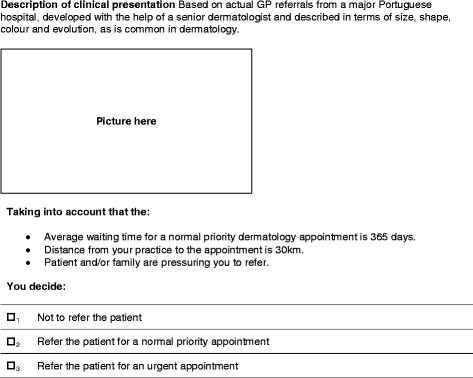
Example of a choice scenario.

The combination of all levels of attributes resulted in 4x4x4x2 = 128 choice situations. In other words, situations in which the GP needs to decide whether or not to refer, given a specific clinical presentation, waiting time, distance, and pressure from patients. Naturally, it would be infeasible to present 128 scenarios to each GP, so we created 8 questionnaire versions with 16 scenarios each, making sure that all levels and attributes appeared with equal frequency across versions (this is commonly done in DCEs and is referred to as blocking). To test for GP heterogeneity, one choice scenario was repeated across all versions, so that 7 versions had 17 scenarios (GP heterogeneity is not considered in this study but could be in future work). An example of a choice scenario – as presented to respondents – is provided in Figure 1 (the format is the same for all scenarios except the levels change). While we asked GPs what priority they would choose for referrals (see Figure 1), we do not use that information in this analysis (i.e., we aggregate all normal priority and urgent referrals).

On the first page of each questionnaire, before the choice scenarios, GPs were asked to provide information on their age, gender, years of experience, number of patients in their practice, region, distances to the closest private and public dermatologists, an assessment of their patients’ general health status, whether they had a special interest in dermatology, and finally whether they had participated in real-time teleconsultations. Responses to these questions provided further information to characterise referral preferences. Altogether, the questionnaire provided us with multiple variables from all four categories identified in the literature.

Around 600 self-complete questionnaires were distributed in two meetings of the Portuguese Association of General Practice, in early 2013. Questionnaires were included in the delegates’ welcome packages which were distributed on a first-come-first-served basis, so that respondents were randomly assigned to one of the 8 versions. Based on the pre-test, questionnaires took an average of 8 minutes to complete.

### Model specification and data analysis

For each of the 16 scenarios in each questionnaire, respondents were asked whether or not they would refer the patient. The answer is thus a binary variable (yes or no, 1 or 0) and so generalised linear regression techniques must be used. We used a binary logistic regression model to predict the outcome of the dependent variable (modelled as Y = 1 if a referral was made and Y = 0 otherwise) based on the values of the independent, or explanatory, variables (e.g., clinical presentation, GP age, etc.). As well as a general error term capturing unobserved variation across GPs, we also included a GP-specific error term to account for the fact that there were multiple observations for each GP. Two models were estimated: model 1a with only attributes and model 1b with both attributes and covariates. This allowed us to test for changes in the sign, magnitude, and statistical significance of explanatory variables, which could indicate confounding.

After running the logistic model, marginal effects were used to determine how the probability of a referral being made is associated with changes to specific attributes or characteristics. To determine the association between participation in teleconsultations and the probability of a referral being made, we calculated marginal effects at representative values (i.e., all other explanatory variables were set to their sample means or modes) for each of the four clinical presentations. All statistical analyses were performed with Stata 12.

The study was sponsored by the Portuguese *Fundação para a Ciência e a Tecnologia* and conducted in Portugal. The sponsor does not require ethical approval for anonymous surveys (no identifiable data was collected) which do not involve patients, as was the case. Participation in the study was voluntary and no incentives were offered.

## Results

Responses from 44 GPs were included in the study, giving a total of 721 usable observations. It is not possible to determine the response rate since we do not have the exact number of participants who received questionnaires (some delegates did not show up to the meetings), although a conservative estimate would be 44 out of 600, or 7.3%. Six GPs did not answer all cases (the minimum number of responses was 11) and for a number of explanatory variables (age, practice size, and distance to closest private dermatologist) there were some missing values. The characteristics of the sample are shown in Table 1.

**Table 1:** **Characteristics of the sample**

**Characteristic**	**n** ^*1^	**Mean**	**SD** ^*2^
*Gender*			
Male	7 (16)		
Female	37 (84)		
*Participation in teleconsultation*			
Yes	11 (25)		
No	33 (75)		
*Special interest in dermatology*			
Yes	29 (67)		
No	15 (33)		
*General health status of patients*			
Very good	-	-	
Good	1 (2)		
Neither good nor bad	20 (46)		
Bad	22 (50)		
Very bad	1 (2)		
*Age*	42 (95)	36	11
*Years of experience*	44 (100)	9	10
*Distance to public dermatologist (km)*	44 (100)	16	19
*Distance to private dermatologist (km)*	30 (68)	6	13
*Number of patients in practice*	43 (98)	22,092	26,276

^*1^N = 44, percentages in parenthesis, values lower than 100% indicate missing values;^*2^standard deviation.

Across all 721 choice situations, the average referral rate (i.e., the number of referrals divided by the total number of cases) was 72.0%. Across GPs, the lowest referral rate was 47.1% and the highest 100%. Indeed, two GPs chose to refer every case in their questionnaires. The average referral rates for each clinical presentation were: 96.2% for melanoma; 97.6% for melanocytic naevus; 28.2% for seborrhoeic keratosis; and 60.5% for psoriasis. Variation was highest for seborrhoeic keratosis and psoriasis, with a number of GPs referring every case and some referring none.

### Association between participation in teleconsultation and referral rates

Of the 44 GPs who returned questionnaires, 11 (25%) had participated in teleconsultations. Across all clinical presentations, the average referral rate for those 11 GPs was 68.1%, compared to a rate of 74.4% for the remaining GPs who had never participated in teleconsultations (see Table 2). The range (i.e., difference between the maximum and minimum referral rates) for GPs who had participated in teleconsultations was 33% lower than the range for the remaining GPs. This is also visible in the lower standard deviation.

**Table 2:** **Referral rates of participating and non-participating GPs: descriptive statistics**

	**Mean**	**Median**	**Min**	**Max**	**Range** ^*1^	**SD** ^*2^
Referral rate of participating GPs	0.68	0.65	0.53	0.88	0.35	0.11
Referral rate of non-participating GPs	0.74	0.76	0.47	1.00	0.53	0.15

^*1^Difference between max and min; ^*2^standard deviation.

In the logistic regression (results shown in Table 3), the explanatory variable capturing whether a GP had participated in teleconsultations was negative and highly significant (p = 0.002) indicating that participation in teleconsultations is associated with a lower likelihood of a referral being made, holding all other independent variables constant. The need variables (melanoma, naevus and psoriasis) were all highly significant and positive, indicating that GPs were more likely to refer patients presenting with these conditions compared to keratosis (the base case). Non-clinical attributes (waiting time, distance and pressure from patient) were not statistically significant. Physicians from practices farther away from the referral hospital were more likely to refer. Both age and age-square were statistically significant suggesting age affects the likelihood of a referral being made in a non-linear way (years of experience was highly correlated with age and thus excluded). Those GPs with a special interest in dermatology were generally less likely to refer.

**Table 3:** **Results of logit models of decision to refer/not refer**

**Variables**	**Model 1a**	**Model 1b**
Intercept	−1.062***	−13.12***
	(0.371)	(4.503)
Nevus	5.008***	5.901***
	(0.474)	(0.737)
Melanoma	5.549***	5.409***
	(0.598)	(0.680)
Psoriasis	1.746***	1.761***
	(0.279)	(0.358)
Waiting time	0.000184	0.0000828
	(0.000938)	(0.00118)
Distance	−0.00403	−0.00286
	(0.00325)	(0.00411)
Pressure	0.0671	0.219
	(0.246)	(0.316)
Age		0.606**
		(0.245)
Male		−0.186
		(0.650)
Distance hospital		0.0400**
		(0.0162)
Distance private		−0.0293
		(0.0214)
List size		0.0000163
		(0.0000227)
Telemedicine		−1.911***
		(0.603)
Special interest		−1.149**
		(0.526)
Health status: bad		0.423
		(0.481)
Health status: good		−1.698
		(1.377)
Age-squared		−0.00680**
		(0.00293)
*Observations*	721	473
*AIC*	514.1	327.2
*Log likelihood*	−249.1	−145.6
*Chi-squared*	152.5***	95.37***
*Hosmer & Lemeshow Chi-squared*	12.22	13.84*
*% pred. correctly*	82.25%	86.47%
*Area under ROC*	0.8763	0.9215

***significant at 1% level; **significant at 5% level; ^*^significant at 10%. Dependent variable is a dummy variable indicating whether a referral was made. Base categories for explanatory dummy variables: Need – Keratosis; Pressure – No; Telemedicine – No; Special interest – No; Health status – Neither good nor bad; standard errors in parenthesis.

Both the percentage of correctly predicted responses and the area under the receiver operating curve indicate the inclusion of more covariates provided a better fit. The Hosmer & Lemeshow test statistic also confirms goodness of fit (chi-squared not significant). Significant effects and signs of coefficients from model 1a (only attributes) persist in model 1b (attributes and covariates), indicating that estimates are robust to the inclusion of other variables.

Keeping all other variables constant at their sample means or modes, participation in teleconsultations was associated with reductions in the probabilities of referral of: 17.6% for patients presenting with seborrhoeic keratosis (p = 0.02); 42.3% for those presenting with psoriasis (p < 0.001); 8.4% for cases of melanoma (p = 0.14); and 5.4% for patients presenting with naevus (p = 0.19).

## Discussion

The results indicate that GP participation in teleconsultation is associated with overall reductions in referral rates and in variation across GPs. In other words, GPs who participate in teleconsultations not only refer fewer patients, they exhibit more homogeneous rates of referral. The logistic regressions confirm that GP participation in teleconsultation is associated with a reduction in the likelihood of a referral being made and – importantly – that the reduction is robust to the inclusion of other factors in the model (something that is not possible to conclude using only bivariate analyses or frequencies).

As intuitively expected, GPs with a special interest in dermatology had lower rates of referral. In line with previous studies, the different clinical presentations (intended as a gradient of clinical need) had the biggest impact on GPs’ decisions, but non-clinical attributes were also important. While typical access indicators such as waiting time and distance were not statistically significant, the distance from the GP practice to the referral hospital was significant and positive. One interpretation is that GPs from practices farther away from the referral hospital are more likely to be isolated from peers, have fewer patients and are overall more in need of support. If this is the case, it would mean that distance has different effects for patients (where it acts as a barrier to access and thus leads to less utilisation) compared to GPs.

Although pressure from patients and/or family was not statistically significant, previous studies did not explore the impact of patient pressure in the context of dermatology [28]. Finally, we found evidence that GPs’ age was associated with referral rates in a non-linear way. As GPs grow older they are more likely to refer patients, *ceteris paribus*, up to a certain age (around 45 years old), after which the reverse is true. According to previous research, older GPs are more experienced, while younger GPs tend to be more risk averse and uncertain, thus referring more often. The relationship we observe could be a direct result of the age distribution in our sample: there are very few respondents between 38 and 52 years of age. If for some reason these GPs are more likely to refer than the rest, then that would explain our results.

The estimated marginal effects indicate that GP participation in teleconsultations is associated with considerable and statistically significant reductions in the probabilities of referring patients with conditions that should be managed in primary care (keratosis and psoriasis), but statistically insignificant reductions in the probabilities of referring patients presenting with more severe conditions (melanoma and naevus). As such, it is not possible to reject the possibility that the reductions found for melanoma and naevus are, in fact, zero (i.e., the *null hypothesis*). It is important to explore, in the future, whether GPs who have participated in teleconsultations are less likely to refer urgent and severe cases, which would be a cause for concern.

While the regression coefficients indicate that participation in teleconsultation and having a special interest in dermatology are each independently associated with lower referral rates, we explored whether these effects might be confounded. Of the 11 GPs who participated in teleconsultations, 8 (72.7%) had a special interest in dermatology, while only 63.6% (21 out of 33) of non-participating GPs shared that interest. The lowest referral rates were seen for the three GPs who had participated in teleconsultations but did not have a special interest in dermatology (66.7%), followed by the 8 GPs who had participated in teleconsultations and had a special interest in dermatology (68.7%), the 21 GPs who had not participated in teleconsultations but had a special interest in dermatology (70.9%) and finally the 12 GPs who had neither participated in teleconsultations nor had a special interest in dermatology (80.4%). These numbers provide further strength to the hypothesis that participation in teleconsultation is associated with lower referral rates, independently of having an interest in dermatology.

While it is not possible for us to say whether there is a causal relationship between participation in teleconsultations and lower rates of referral, our findings provide multiple indications that GPs who have participated in teleconsultations have lower rates of referral, even after controlling for numerous patient, GP, practice and secondary care factors previously shown to influence referral decisions. The fact that not only the rates but also the range and standard deviation are lower may suggest that some form of education is taking place (otherwise rates would simply be lower across the board) and motivates future research into why we see this association.

### Limitations and future work

A significant limitation of this study is the size and representativeness of the sample of GPs: it is small and mostly composed of young female GPs. Given conflicting results in previous research regarding the impact of age and gender on referral rates, it is not possible to speculate how the sample may have biased the results. While the findings are informative of a younger predominantly female GP population, they may not be generalizable to the larger population of Portuguese GPs, or indeed to GPs in other countries. Furthermore, the fact that only 44 GPs returned questionnaires means there were only 44 values for a number of explanatory variables (e.g., age, practice size, etc.). This limited the complexity of specifications available to us and made it infeasible to model interactions. Future work should seek to achieve bigger sample sizes and to conduct similar studies in other countries besides Portugal.

Even though we were able to simultaneously include multiple factors in our analyses, there are a number of variables for which we have no information. We cannot exclude the possibility that the physicians in our sample who participated in teleconsultations were also more dedicated to improving care and using a variety of educational tools other than teleconsultations. We have also not collected data pertaining to the frequency, conditions and specialties of teleconsultations, limiting our understanding of why participation in teleconsultations was associated with lower rates of referral. For example, while participation in teledermatology appointments might justifiably be associated with lower rates of referral to dermatologists, it is unclear why participation in teleneurology or telecardiology appointments might also lower referrals to dermatologists.

Finally, a consequence of using self-reported data is that external validity may be an issue. It is healthy to be sceptical about relying on what people say they will do compared to what they actually do [29]. One way to test external validity would be to combine data from this study with observational data on actual referrals made by respondents. This would however require respondents to be identified, potentially leading to selection bias (GPs would self-select into the sample). This possibility could be explored in the future.

## Conclusions

Even taking into account the limitations, there are reasons to be confident in the study’s findings. First, the coefficients in the logistic regression are in line with previous literature and with expectations. And second, the tests of specification and fit indicate the model is well-specified and provides a good fit. This study shows that DCEs can be used to explore the association between GP participation in teleconsultations and referral rates, but it should be seen as a first step. With bigger samples from multiple settings, more sophisticated models of referrals can be used, accounting for more explanatory variables, interactions (i.e., the possibility that there are mediator variables), and parameters (i.e., the prospect that different GPs are affected differently, something that is commonly referred to as respondent heterogeneity). Perhaps most importantly, future studies should explore why GP participation in teleconsultations is associated with lower referral rates, and whether this is the result of a causal relationship involving continuing education and GP learning.

### Availability of supporting data

Supporting data not made available.

## Abbreviations

DCE: Discrete choice experiment
GP: General practitioner
ROC: Receiver operating curve
